# Impact of Hypoglycemia on Health-Related Quality of Life among Type 2 Diabetes: A Cross-Sectional Study in Thailand

**DOI:** 10.1155/2019/5903820

**Published:** 2019-10-23

**Authors:** Thongchai Pratipanawatr, Bancha Satirapoj, Boonsong Ongphiphadhanakul, Sompongse Suwanwalaikorn, Wannee Nitiyanant

**Affiliations:** ^1^Department of Medicine, Faculty of Medicine, Srinagarind Hospital, Khon Kaen University, Khon Kaen 40002, Thailand; ^2^Department of Medicine, Phramongkutklao Hospital and College of Medicine, Bangkok 10400, Thailand; ^3^Department of Medicine, Faculty of Medicine, Ramathibodi Hospital, Mahidol University, Bangkok 10400, Thailand; ^4^Department of Medicine, Faculty of Medicine, King Chulalongkorn Memorial Hospital, Chulalongkorn University, Bangkok 10330, Thailand; ^5^Department of Medicine, Siriraj Hospital, Bangkok, Thailand

## Abstract

Type 2 diabetes mellitus (T2DM) is one of the most common chronic diseases. Patients are generally advised lifestyle changes with antihyperglycemic agents prescribed. The major drawback of prescribing antihyperglycemic agents is the risk of hypoglycemia which subsequently impacts on health-related quality of life (HRQoL). This study is aimed at examining association between previous history of hypoglycemia and HRQoL. The study was a multicenter cross-sectional study, conducted from February 2013 to March 2015 at 5 tertiary care hospitals in Thailand (Srinagarind, Phramongkutklao, Ramathibodi, King Chulalongkorn Memorial, and Siriraj hospitals). The study population were males or females diagnosed with type 2 DM according to ADA criteria, 30 years of age or older, who had been treated with sulfonylurea (SU) monotherapy or SU and metformin combination for at least 6 months. Prespecified medical factors were extracted from medical records 12 months prior to patients' enrolment. The experience of hypoglycemia questionnaire was used to collect and measure severity of hypoglycemia experienced during the previous 6 months. HRQoL was assessed using the 3-level version of EuroQol-5-dimension (EQ-5D-3L) and visual analogue scale (EQ-VAS) questionnaires. Of 659 eligible patients surveyed, 202 patients (30.65%) had experienced symptoms of hypoglycemia. HRQoL was significantly lower among patients reporting at least one of hypoglycemic symptoms, measured by EQ-VAS scores (mean ± SD; 73.66 ± 13.18, 73.56 ± 15.10, or 68.93 ± 14.76 vs. 77.01 ± 13.02, one-way ANOVA; *p* = 0.006) and EQ-5D-3L index scores (0.62 ± 0.47, 0.68 ± 0.38, or 0.58 ± 0.51 vs. 0.79 ± 0.31, one-way ANOVA; *p* < 0.001) for mild, moderate, or severe/very severe hypoglycemic patients compared with patients without hypoglycemic symptoms. After adjusting for confounding factors in a multiple linear regression model, patients with hypoglycemic symptoms either mild, moderate, or severe/very severe demonstrated significantly higher impairment for EQ-VAS and EQ-5D indexes than those who did not experience hypoglycemic symptoms. In conclusion, our study showed decreased HRQoL determined by EQ-5D and EQ-VAS in patients reporting symptoms of hypoglycemia compared with patients not reporting hypoglycemic symptoms, relative to severity of hypoglycemia.

## 1. Introduction

Type 2 diabetes mellitus (T2DM) is a chronic and progressive disease with serious long-term micro- and macrovascular complications [1] resulting in an increase in morbidity, mortality, healthcare costs, and a decrease in HRQoL. There are several treatments for diabetes, with the most common treatment regimen being the prescription of oral antihyperglycemic agents and insulin therapy which consequently induce hypoglycemia. The most prominent hypoglycemia inducers are sulfonylureas and insulin therapy. Hypoglycemia can be a major barrier to achieving treatment goals and has been shown to increase risks of cardiovascular events [2], dementia [3], fall-related fracture [4], and “defensive eating” which contributes to weight gain. In addition, patients' apprehension about hypoglycemia can also be a major implication in disease management [5]. The particular impact of hypoglycemia on health-related quality of life (HRQoL) has often been demonstrated in observational studies [6, 7], suggesting the indirect correlation between hypoglycemia induced from diabetes treatments on HRQoL. Nonetheless, the hypoglycemic symptoms seen in diabetic patients may also be influenced by other factors including old age, weight gain, comorbidity and cardiovascular disease, gender differences, types of antihyperglycemic agents taken such as metformin, concerns about hypoglycemia, and overall patients' satisfaction, which in-turn may also affect patients' quality of life. Therefore, the association of hypoglycemia with HRQoL is inconclusive as there are other confounding factors involved. This study is aimed at examining the association between previous history of hypoglycemia and HRQoL in Thai T2DM patients.

## 2. Materials and Methods

### 2.1. Study Design and Setting

This study was a multicenter cross-sectional study conducted in patients who have been treated with sulfonylurea (SU) monotherapy or SU and metformin combination therapy (SU+MET) for at least 6 months prior to the study enrolment in 5 tertiary care hospitals in Thailand (i.e., Srinagarind, Phramongkutklao, Ramathibodi, King Chulalongkorn Memorial, and Siriraj hospitals). The study was conducted from February 2013 to March 2015. This study was approved by the Ethics Committee of each hospital. The potential patient medical charts were retrospectively reviewed to check if they meet inclusion and exclusion criteria. The patients satisfying the selection criteria were enrolled in the study after providing written informed consent to participate in the study.

### 2.2. Participants

The patients who were diagnosed with T2DM according to American Diabetes Association (ADA) criteria [8], 30 years of age or older, and treated with SU monotherapy or SU+MET for at least 6 months before enrolment by an endocrinologist, cardiologist, nephrologist, or family practitioner were included, whereas the patients who were pregnant; have Type 1 DM; required daily concomitant insulin; received other oral hypoglycemic agents, except for SU or SU combined with metformin; were participating in other clinical trial studies; and were not able to complete the study questionnaires were excluded from this study.

### 2.3. Study Measurements and Study Data Collection

A standardized data collection form was developed to record data of clinical and laboratory assessment of patients from medical records that were completed by physicians or personnel who had been trained. Prespecified medical data from charts were extracted for the 12-month period prior to the patient enrolment date. On the day of enrolment, participating patients were subjected to a standard blood draw after overnight fasting for measurements of HbA_1c_, fasting plasma glucose, serum creatinine, total cholesterol, triglycerides, HDL-cholesterol, and LDL-cholesterol. In addition, each patient's body weight, blood pressure, and waist circumference were measured and recorded. Alcohol consumption and regular physical activity were also assessed.

Experience of hypoglycemia during the previous 6 months was assessed with the questionnaire that was developed by Merck Sharp & Dohme (MSD) (see the Experience of Low Blood Sugar (Hypoglycemia) questionnaire in Supplementary [Supplementary-material supplementary-material-1]). The hypoglycemia symptoms were stratified by severity (from none, mild, moderate, and severe to very severe). The patients were subsequently classified according to having experienced hypoglycemia or not (yes/no) and according to the maximum severity of hypoglycemic episodes experienced.

HRQoL was assessed by using the Thai language 3-level version of EuroQol 5-dimension (EQ-5D-3L) questionnaire which is a standardized instrument for measuring the general health outcome and is a recommended utility method because it has acceptable feasibility and validity in Thailand [9]. The EQ-5D contains 5 items to be answered using a 3-point rating scale plus a visual analogue scale (VAS) from 0 (worst imaginable health state) to 100 (best imaginable health state) [10]. The EQ-5D measures 5 dimensions of health: mobility, self-care, usual activities, pain/discomfort, and anxiety/depression. The EQ-5D index score was calculated using the coefficient from the “Social tariff for EuroQol” [11].

The treatment satisfaction was evaluated by using the 14-item treatment satisfaction questionnaire for medication (TSQM, version 1.4) [12]. The satisfaction score per dimension was calculated ranging from 0 to 100, with a lower score expressing a better treatment satisfaction. Worry about hypoglycemia and self-reported adherence were assessed by the Worry Scale of HFS II [13, 14] and the Self-Report Adherence and Barriers Questionnaire [15], respectively. The questionnaires were previously translated from English to Thai and used in a diabetes study (the Asia-Pacific Real-Life Effectiveness and Care Patterns of Diabetes Management (AP RECAP-DM) study) [16]. The authors also reviewed and verified the accuracy of the translations prior to use in this study.

### 2.4. Sample Size

We estimated the sample size in order to conduct our survey of HRQoL in hypoglycemia patients using the following formula [17]: *n* = (*Z*^2^ × *P*(1–*P*))/*d*^2^. The expected prevalence of hypoglycemia was 36% which was reported in the AP RECAP-DM study (Poster No. P44, 7^th^ IDF Western Pacific Region Congress, Wellington, New Zealand, March 30-April 3, 2008). The sample size was calculated assuming the statistic corresponding level of confidence of 95% (*Z*, 1.96), hypoglycemia proportion of 0.36, and a desired margin of error (*d*) of ±3.5% to 4%. Therefore, we planned to study HRQoL in approximately 600 to 723 eligible patients.

### 2.5. Statistical Analysis

Descriptive statistics were used to report demographic characteristics of patients and clinical characteristics, including patient sociodemographic, clinical, and laboratory test results. The Shapiro-Wilk test was used for checking normality of the continuous data. If the data distribution is normal, the data will be presented by the mean (±SD). If the data significantly deviate from a normal distribution (*p* value < 0.05), the data will be presented by the median (IQR). The demographic characteristics, laboratory results, and HRQoL of patients with and without reported hypoglycemic symptoms were compared using the Chi-squared test or Fisher's exact test for categorical variables and independent sample *t*-test or Mann-Whitney *U* test for continuous variables.

One-way analysis of variance (one-way ANOVA) was conducted to evaluate differences in EQ-VAS scores by severity of hypoglycemia episodes and evaluated differences in EQ-5D-3L index scores by episode severity of hypoglycemia. Multivariate linear regression, adjusting for potential confounders, was used to estimate the effect of hypoglycemia on HRQoL (EQ-VAS and EQ-5D-index scores). The technique for selecting the covariates in the multivariate regression analyses was that based on the directed acyclic graph (DAG). Multivariate relationships were conceptualized using directed acyclic graphs and minimum sets of adjustment variables to obtain unbiased estimates of total and direct effects of various exposure variables on occurrence of hypoglycemia, treatment compliance, treatment satisfaction, quality of life, worry about hypoglycemia, and fear of weight gain compatible with the conceptual graph identified.

Directed acyclic graphs were constructed using DAGitty software (version 2.3) [18]. All test statistics applied a *p* value less than 0.05 as statistically significant. The study data analyses were performed using STATA release 14.1 (StataCorp, College Station, TX).

## 3. Results

### 3.1. Participants

**Figure 1:**
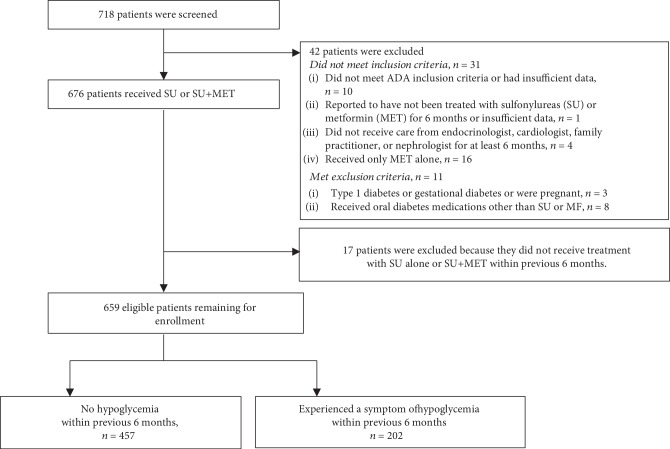
Participant flow.

Participant flow is shown in Figure 1. Approximately 30% (202 of 659) of type 2 diabetes patients experienced symptoms of hypoglycemia in 6 months prior to the survey.

Compared to those who did not experience symptoms of hypoglycemia, patients who did experience symptoms of hypoglycemia were significantly younger (mean ± SD, 63.94 ± 10.65 years vs. 66.20 ± 9.59 years, *t*-test; *p* = 0.008) and less commonly took regular physical activities (no regular activity rate; 34.3% vs. 42.8%, Chi-squared test; *p* = 0.038). In addition, the patients experiencing hypoglycemia were more commonly on a low-sugar diet higher than those who did not experience symptoms of hypoglycemia (57.7% vs. 47.6%, Chi-squared test; *p* = 0.018) and were more commonly nondrinkers (74.5% vs. 69.6%, Chi-squared test; *p* = 0.030).

There were no significant differences in waist circumference, systolic and diastolic blood pressure, micro-/macrovascular complications, and hypoglycemic agents used between the patients with hypoglycemia and without hypoglycemia. In addition, there were no significant differences in marital status, education, or having parents with diabetes mellitus (data not shown). There were no significant differences between patients taking SU versus patients taking SU+MET in terms of hypoglycemia symptoms experienced. The patient demographics by occurrence of hypoglycemia in the previous 6 months are shown in Table 1. Furthermore, there were also no significant differences in respect to the laboratory test results between patients who did and did not experience hypoglycemic symptoms (Table 2).

### 3.2. Hypoglycemia and EQ-VAS and EQ-5D Index Scores

HRQoL was significantly lower among patients reporting at least one of hypoglycemic symptoms either mild, moderate, or severe/very severe symptoms, as measured by EQ-VAS scores (mean ± SD; 73.66 ± 13.18, 73.56 ± 15.10, or 68.93 ± 14.76 vs. 77.01 ± 13.02, one-way ANOVA; *p* = 0.006) and EQ-5D index scores (0.62 ± 0.47, 0.68 ± 0.38, or 0.58 ± 0.51 vs. 0.79 ± 0.31, one-way ANOVA; *p* < 0.001) compared to patients reporting no symptoms as shown in Table 3.

### 3.3. Hypoglycemia and EQ-5D Subscores

There was significant association between quality of life in each domain and severity of hypoglycemic episodes experienced as shown in Table 4.

### 3.4. Association between Severity of Hypoglycemic Episodes and HRQoL

The results show that higher severity of hypoglycemic episodes experienced, use of SU+MET, higher worry about hypoglycemia scores, weight changes (either lost or gained), and higher fear of weight gain scores were significant factors lowering EQ-5D-3L index score while being male significantly increased the score. The patients with higher severity of hypoglycemic symptoms were negatively associated with HRQoL as measured by EQ-5D-3L index score than those who did not have hypoglycemia (adjusted mean difference; for mild: -0.156 (95% CI: -0.225, -0.087), moderate: -0.096 (CI: -0.183, -0.008), and severe/very severe: -0.198 (CI: -0.373, -0.023) when compared to those who have not experienced hypoglycemia).

For HRQoL, as measured by EQ-VAS scores, higher severity of hypoglycemic episodes experienced and higher worry about hypoglycemia were significant factors lowering EQ-VAS score while higher overall satisfaction was related with a higher EQ-VAS score. The patients with higher severity of hypoglycemic episodes experienced symptoms also demonstrated significantly higher impairment for EQ-VAS score with adjusted mean difference of -2.92 (95% CI: -5.61, -0.23), -3.17 (95% CI: -6.59, 0.25), and -7.89 (95% CI: -14.71, -1.07) for mild, moderate, and severe/very severe hypoglycemia symptoms when compared to those who have not experienced hypoglycemia.  Table 5 presents the adjusted mean differences of EQ-5D-3L index and EQ-VAS scores of each factor.

## 4. Discussion

In this study of Thai patients with T2DM treated with SU alone or SU+MET, symptoms of hypoglycemia were reported by approximately 30.65% of patients and only 6.59% reported severe or very severe events. The proportion of patients reporting hypoglycemic symptoms reported by United State (US) patients (63% during 6 months [19]) and European patients (38% during the past year [20] and 73% during 3 months [21]) treated with oral antihyperglycemic agents were higher than our study. However, a study in which we found a high prevalence of any form of reported hypoglycemia was in an urban African-American population with type 2 diabetes which reported lower proportion (16%) for those using oral agents alone [22].

Our results demonstrated that patients with hypoglycemic symptoms reported a decrease in EQ-5D index and EQ-5 VAS scores relative to patients who did not, even after adjusting for a number of confounders. This is similar to the results published in Marrett et al. [23], Williams et al. [6], Gilet et al. [24], and Shi et al. [25] which also used the EQ-5D and observed that self-reported hypoglycemia was associated with decrease in adjusted EQ-5D scores [23]. Our results also showed a relationship between the severity of hypoglycemia and decrease in EQ-5D index and EQ-VAS, emphasizing the effect of hypoglycemia on quality of life. Patients who experienced hypoglycemic symptoms reported decreases in all 5 EQ-5D domains. There are more limitation on self-care domain in our study, which were different from the study by Williams et al., in which self-care activities were not significantly different between the hypoglycemia severity groups [6]. These study results are similar to the results of Marrett et al. [23], which also used EQ-5D and found that the adjusted mean EQ-5D index score (reference = no hypoglycemia) was significantly 0.045 lower for those reporting any hypoglycemic symptoms and by symptom severity: mild 0.009, moderate 0.055, severe 0.131, and very severe 0.208 [23]. Our results also show that the worry about hypoglycemia decreases both EQ-5D index and EQ-VAS scores. Therefore, therapies, patient monitoring rationales, or patient education programs that minimize the frequency and severity of hypoglycemia and worry about hypoglycemia would likely increase the diabetic patients' quality of life.

The current results should be considered within the context of several limitations. Hypoglycemia was determined by a patient-reported low plasma glucose questionnaire, cross-sectional survey. The hypoglycemia data were not verified against clinician diagnoses or chart reviews nor were reports of low blood sugar confirmed by blood glucose monitoring. The future studies of quality of life at the time or immediately after the hypoglycemia symptom confirmation or when the hypoglycemia is confirmed by blood sugar monitoring are needed. In addition, this study is limited by its cross-sectional design. Future studies with more rigorous designs, such as prospective cohort studies, are also needed to confirm the impact of hypoglycemia and to compare within the individual patients.

Additional consideration is that EQ-5D index scores were calculated using the coefficient from the “Social tariff for EuroQol” [11], which is based on the perceptions of a sample drawn from the UK population. Quality-of-life perceptions may be different in the Thai population from those in a UK population. However, EQ-VAS score which can directly quantify overall quality of life showed similar results to EQ-5D index score. Although a study that compared between EQ-5D-5L and EQ-5D-3L supported the convergent validity and test-retest reliability of both the 3L and 5L in diabetes patients, the authors recommend to use the 5L due to being more promising in terms of a lower ceiling, more discriminatory power, and higher preference by the respondents compared to the 3L [26]. Therefore, the future study in Thai patients with EQ-5D-5L is recommended.

## 5. Conclusions

In this survey of Thai patients with T2DM who were treated with SU alone or SU+MET, approximately one-thirds reported experiencing hypoglycemic symptoms. The results from the study showed significant decrement of HRQoL as determined by the EQ-5D index scores and the EQ-VAS in the patient-reported symptoms of hypoglycemia compared with patients who did not report those symptoms, relative to severity of hypoglycemia. Even after adjusting for a number of variables, patients who experienced hypoglycemic symptoms still continued to report a decrease in the HRQoL.

## Figures and Tables

**Table 1:** Patient demographics by occurrence of hypoglycemia in previous 6 months.

Variables	No hypoglycemia (*n* = 457)	Hypoglycemia (*n* = 202)	*p* value
Male, *n* (%)	228 (50.8)	93 (46.0)	0.272^a^
Age (years)	66.20 (9.59)	63.94 (10.65)	0.008^b†^
Duration of DM (years), median (IQR)	10 (5, 15)	10 (6, 15)	0.531^c^
Height (cms)	160.5 (8.4)	160.3 (9.3)	0.746^b^
Body weight (kgs)	66.5 (12.9)	65.2 (14.5)	0.134^b^
Did not take physical regular activity, *n* (%)	156 (34.3)	86 (42.8)	0.038^a†^
Alcohol consumption, *n* (%)			
Never	318 (69.6)	149 (74.5)	0.030^a†^
Occasionally	116 (25.4)	45 (22.5)	
Daily	1 (0.2)	3 (1.5)	
Unknown	22 (4.8)	3 (1.5)	
Low-sugar diet, *n* (%)	214 (47.6)	116 (57.7)	0.018^a†^
Weight change in previous 12 months			
No change	87 (19.3)	42 (21.1)	0.798^a,*β*^
Gained	158 (35.0)	65 (32.7)	
Lost	192 (42.5)	85 (42.7)	
Unknown	15 (3.3)	7 (3.5)	
Waist circumference (cm)	92.4 (10.1)	91.0 (11.7)	0.119^b^
SBP (mmHg)	135.7 (17.1)	133.5 (17.6)	0.128^b^
DBP (mmHg)	74.5 (10.2)	73.4 (9.8)	0.186^b^
Hypoglycemic agents, *n* (%)			
Sulfonylurea (SU)	93 (20.3)	45 (22.3)	0.604^a^
Combination of SU and metformin	364 (79.7)	157 (77.7)	
Macro- and/or microvascular complications, *n* (%)	99 (21.7)	38 (18.8)	0.407^a^

^a^Chi-squared test or Fisher exact test as appropriate; ^b^independent sample *t*-test; ^c^Mann-Whitney *U* test. † indicates statistically significant difference between the occurrence of hypoglycemia groups (*p* value < 0.05). ^*β*^Only known data used for analysis. Data are the mean ± standard deviation (SD) unless otherwise specified. Abbreviation: SD: standard deviation; IQR: interquartile range.

**Table 2:** Laboratory test results at enrolment by occurrence of hypoglycemia in the previous 6 months.

Variables	No hypoglycemia (*n* = 457)	Hypoglycemia (*n* = 202)	*p* value
HbA_1c_ (%)	7.29 (1.28)	7.17 (1.31)	0.247^a^
FPG (mg/dL)	145.6 (44.6)	139.4 (39.7)	0.086^a^
Serum creatinine (mg/dL)	1.26 (1.08)	1.23 (0.89)	0.767^a^
Total cholesterol (mg/dL)	167.8 (38.5)	170.3 (36.1)	0.581^a^
HDL-cholesterol (mg/dL)	50.4 (20.4)	51.1 (15.0)	0.784^a^
LDL-cholesterol (mg/dL)	97.7 (33.9)	98.1 (32.4)	0.912^a^
Triglycerides (mg/dL), median (IQR)	133 (92, 179)	132.5 (91.5, 180)	0.632^b^
Urinary albumen (mg/g), median (IQR)	10.8 (4.7, 30.3)	7.3 (2.3, 46.4)	0.573^b^

^a^Independent sample *t*-test. ^b^Mann-Whitney *U* test. Data are the mean ± standard deviation (SD) unless otherwise specified. Abbreviation: HbA_1c_ = hemoglobin A1c; FPG = fasting plasma glucose.

**Table 3:** Patient EQ-5D-VAS and EQ-5D-index scores according to severity of hypoglycemia.

HRQoL	Maximum severity of hypoglycemic episodes experienced	Mean (SD)	95% CI	*p* value^∗^
EQ-5D-VAS score	None	77.01 (13.02)	75.77, 78.22	0.006
	Mild	73.66 (13.18)	71.27, 76.06	
	Moderate	73.56 (15.10)	70.37, 76.45	
	Severe/very severe	68.93 (14.76)	62.18, 75.68	

EQ-5D index score	None	0.79 (0.31)	0.76, 0.82	<0.001
	Mild	0.62 (0.47)	0.56, 0.69	
	Moderate	0.68 (0.38)	0.60, 0.76	
	Severe/very severe	0.58 (0.51)	0.40, 0.75	

^∗^One-way ANOVA. EQ-VAS score ranges from 0 to 100, with a higher score indicating better quality of life. EQ-5D index score was calculated using the coefficient from “Social tariff for EuroQol” [11].

**Table 4:** Patient EQ-5D-3L levels by maximum severity of hypoglycemic episodes.

Domain	Maximum severity of hypoglycemic episodes	*n*	Level of perceived problem, *n* (%)			*p* value^∗^
			No problem	Some problem	Extreme problems	
Mobility	None	457	297 (65.0)	140 (30.6)	20 (4.4)	0.002^†^
	Mild	119	52 (43.7)	55 (46.2)	12 (10.1)	
	Moderate	67	35 (52.2)	28 (41.8)	4 (6.0)	
	Severe/very severe	15	9 (60.0)	5 (33.3)	1 (6.7)	

Self-care	None	457	383 (83.8)	71 (15.5)	3 (0.7)	0.001^†^
	Mild	119	80 (67.2)	35 (29.4)	4 (3.4)	
	Moderate	67	54 (80.6)	12 (17.9)	1 (1.5)	
	Severe/very severe	15	10 (66.7)	4 (26.7)	1 (6.7)	

Usual activities	None	457	368 (80.5)	80 (17.5)	9 (2.0)	0.001^†^
	Mild	119	76 (63.9)	39 (32.8)	4 (3.4)	
	Moderate	67	48 (71.6)	16 (23.9)	3 (4.5)	
	Severe/very severe	15	9 (60.0)	4 (26.7)	2 (13.3)	

Pain/discomfort	None	457	265 (58.1)	166 (36.4)	25 (5.5)	0.008^†^
	Mild	119	50 (42.4)	54 (45.8)	14 (11.9)	
	Moderate	67	28 (41.8)	32 (47.8)	7 (10.4)	
	Severe/very severe	15	6 (40.0)	7 (46.7)	2 (13.3)	

Anxiety/depression	None	457	341 (75.1)	102 (22.5)	11 (2.4)	<0.001^†^
	Mild	119	64 (54.2)	42 (35.6)	12 (10.2)	
	Moderate	67	35 (52.2)	26 (38.8)	6 (9.0)	
	Severe/very severe	15	7 (46.7)	4 (26.7)	4 (26.7)	

^∗^Chi-squared test. Numbers may not add up to the total because of missing data. † indicates statistically significant difference (*p* < 0.05).

**Table 5:** Factors associated with health-related quality of life measured by EQ-5D-3L index and EQ-VAS scores from multivariate regression analyses.

Variables^∗^	Adjusted mean difference (95% CI) of EQ-5D-3L index score	*p* value	Adjusted mean difference (95% CI) of EQ-5D-3L VAS score	*p* value
Hypoglycemia				
None	Reference	<0.001^†^	Reference	0.013^†^
Mild	-0.156 (-0.225, -0.087)		-2.92 (-5.61, -0.23)	
Moderate	-0.096 (-0.183, -0.008)		-3.17 (-6.59, -0.25)	
Severe/very severe	-0.198 (-0.373, -0.023)		-7.89 (-14.71, -1.07)	
Age ≥ 60 years	-0.057 (-0.118, 0.003)	0.064	-0.32 (-2.63, 1.99)	0.784
Male	0.108 (0.054, 0.161)	<0.001^†^	1.48 (-0.57, 3.53)	0.156
Vascular complication	-0.020 (-0.046, 0.087)	0.547	-2.24 (-4.77, 0.29)	0.082
Treatment				
SU alone	Reference	0.049^†^	Reference	0.127
SU+MET	-0.067 (-0.133, 0.000)		1.97 (-0.56, 4.51)	
Weight change				
None	Reference	0.003^†^	Reference	0.809
Gain	-0.116 (-0.189, -0.042)		0.83 (-2.05, 3.71)	
Loss	-0.119 (-0.190, -0.048)		0.88 (-1.92, 3.67)	
Adherence to medication	-0.018 (-0.071, 0.034)	0.490	-0.45 (-2.49, 1.60)	0.668
Worry about hypoglycemia/unit score (0 to 4)	-0.059 (-0.103, -0.015)	0.009^†^	-3.14 (-4.83, -1.46)	<0.001^†^
Fear of weight gain/unit score (0 to 4)	-0.070 (-0.099, -0.041)	<0.001^†^	-0.73 (-1.85, 0.39)	0.203
Overall satisfaction/unit score (0 to 100)	0.0016 (-0.0008, 0.0039)	0.184	0.103 (0.014, 0.193)	0.024^†^

^∗^Variables included in multivariate regression analyses. Wald test, *p* = *p* value. † indicates statistical significance at *p* < 0.05.

## Data Availability

The patient-level data used to support the findings of this study have not been made publicly or shared with the third parties due to the institutes/hospitals' Human Research Ethics Committee regulations.

## Supplementary Materials

Supplementary Materials The study questionnaires (i.e., Experience of Low Blood Sugar, Worry Scale of HFS II, TSQM, version 1.4) were translated from English to Thai by the Psychology Department, Faculty of Liberal Arts, Thammasat University. The translations were done by forth and back translation. Content validity was checked using double-blind fashion. The questionnaires were tested by 15 diabetic patients, and the average duration to complete the questionnaire was 41.25 minutes.
