# Supplementary material for: Impact of Hypoglycemia on Health-Related Quality of Life among Type 2 Diabetes: A Cross-Sectional Study in Thailand
Source: J Diabetes Res. 2019 Oct 23;2019:5903820. doi: 10.1155/2019/5903820 (PMC6854960; doi:10.1155/2019/5903820)
Supplement: Supplementary Materials — The study questionnaires (i.e., Experience of Low Blood Sugar, Worry Scale of HFS II, TSQM, version 1.4) were translated from English to Thai by the Psychology Department, Faculty of Liberal Arts, Thammasat University. The translations were done by forth and back translation. Content validity was checked using double-blind fashion. The questionnaires were tested by 15 diabetic patients, and the average duration to complete the questionnaire was 41.25 minutes. [file 5903820.f1.docx]

Experience of Low Blood Sugar (Hypoglycemia)

Below is a list of symptoms you might experience when you have an episode (incident) of hypoglycemia (low blood sugar). Before answering the questions please read the list of symptoms carefully.

Some symptoms of **low blood sugar** (hypoglycemia) are:

- sweating - confusion/feeling disoriented

- shakiness - clumsy or jerky movements

- dizziness - sudden moodiness or behavior changes

- hunger - tingling sensations around the mouth

- headache - difficulty concentrating

- pale skin color - blood sugar is ≤ 70 mg/dL

1. Have you ever felt symptoms of low blood sugar (as described in the box above) in the last 6 months?

_1_ Yes

_0_ No (If no, go to questionnaire HFS)

If **YES**, please tick the box that best describes how severe and how often the symptoms of low blood sugar have been during the last 6 months.

2a. During the last 6 months, did you experience **MILD** symptoms of low blood sugar defined as *Little or no interruption of your activities, and you didn’t feel you needed assistance to manage your episode(s) of low blood sugar or symptoms*?

_1_ Yes

_0_ No

2b. How often have you experienced **MILD** symptoms of low blood sugar?

_0_ I did not experience MILD symptoms of low blood sugar

_1_ 1 to 2 times over the last 6 months

_2_ 3 to 6 times over the last 6 months

_3_ more than once per month

_4_ more than once per week

_5_ everyday

3a. During the last 6 months, did you experience **MODERATE** symptoms of low blood sugar defined as *Some interruption of your activities, but didn’t feel you needed assistance to manage your episode (s) of low blood sugar or symptoms*?

_1_ Yes

_0_ No

3b. How often have you experienced **MODERATE** symptoms of low blood sugar?

_0_ I did not experience MODERATE symptoms

_1_ 1 to 2 times over the last 6 months

_2_ 3 to 6 times over the last 6 months

_3_ more than once per month

_4_ more than once per week

_5_ everyday

4a. During the last 6 months, did you experience **SEVERE** symptoms of low blood sugar defined as *Felt that you needed the assistance of others to manage your episode(s) of low blood sugar or symptoms (for example, to bring you food or drink*)?

_1_ Yes

_0_ No

4b. How often have you experienced **SEVERE** symptoms of low blood sugar?

_0_ I did not experience SEVERE symptoms

_1_ 1 to 2 times over the last 6 months

_2_ 3 to 6 times over the last 6 months

_3_ more than once per month

_4_ more than once per week

_5_ everyday

5a. During the last 6 months, did you experience **VERY SEVERE** symptoms of low blood sugar defined as *Needed medical attention (for example, called an ambulance, visited an emergency room or hospital, or saw a doctor or nurse*)?

_1_ Yes

_0_ No

5b. How many times have you experienced **VERY SEVERE** symptoms of low blood sugar?

|__|__| times during the last 6 months

6. Overall, how much were you bothered by your symptoms of your low blood sugar during the last 6 months?

_0_ Not concerned (I did not have low blood sugar symptoms during the last 6 months)

_1_ Not at all

_2_ A little bit

_3_ Somewhat

_4_ Very

_5_ Extremely
